# A Single Center Study of Genes Involved in Synchronous and Metachronous Multiple Early-Stage Gastric Cancers in Japanese Patients with Current or Former *Helicobacter pylori* Infection

**DOI:** 10.3390/cancers17030464

**Published:** 2025-01-29

**Authors:** Minami Hashimoto, Takuto Hikichi, Reiko Honma, Jun-ichi Imai, Mika Takasumi, Jun Nakamura, Tsunetaka Kato, Takumi Yanagita, Mitsuru Otsuka, Daiki Nemoto, Masao Kobayakawa, Shinya Watanabe, Hiromasa Ohira

**Affiliations:** 1 Department of Endoscopy, Fukushima Medical University Hospital, Fukushima 960-1295, Japan; mi-hashi@fmu.ac.jp (M.H.); junn7971@fmu.ac.jp (J.N.); tsune-k@fmu.ac.jp (T.K.); takumi-y@fmu.ac.jp (T.Y.); nemotoda@fmu.ac.jp (D.N.); mkobaya@fmu.ac.jp (M.K.); 2 Department of Gastroenterology, Fukushima Medical University School of Medicine, Fukushima 960-1295, Japan; paper@fmu.ac.jp (M.T.); m-otsuka@fmu.ac.jp (M.O.); h-ohira@fmu.ac.jp (H.O.); 3 Medicrome Inc., Fukushima 960-8031, Japan; honma.r@medicrome.com; 4 Translational Research Center, Fukushima Medical University, Fukushima 960-1295, Japan; j_imai@mtc.biglobe.ne.jp (J.-i.I.); swata@mvc.biglobe.ne.jp (S.W.); 5 Medical Research Center, Fukushima Medical University, Fukushima 960-1295, Japan

**Keywords:** comprehensive gene expression analysis, gastric cancer, gene expression scoring system, metachronous cancer, multiple cancer

## Abstract

Background: This study aimed to perform a comprehensive gene expression analysis in patients with early-stage gastric cancer (EGC) to identify gene expression profiles specific to gastric cancer (GC) lesions. Methods: Biopsy specimens were collected from one EGC lesion and three background mucosal areas of patients scheduled for endoscopic submucosal dissection (ESD). Lesion-specific gene profiles in these four biopsies were analyzed using DNA microarrays. Patients with concurrent EGCs at the time of an ESD or a history of GC were classified into the multiple GC group (*n* = 26), while those without such histories were assigned to the single GC group (*n* = 74). Results: After excluding patients with heterogeneous factors, 55 patients were analyzed. Twenty-one differential genes exhibiting distinct mean expression profiles stratified by background gastric mucosa were extracted between the single and multiple GC groups. A scoring system constructed using these genes to calculate the weighted expression values for each patient, with an optimal cutoff value of −2.574, yielded a sensitivity and specificity of 85.7%. Conclusions: This study identified the different gene expression profiles between synchronous and metachronous multiple GCs and single GCs in patients with EGC. The developed scoring system has potential to distinguish between single and multiple GCs.

## Simple Summary

This study presents a comprehensive analysis of gene expression profiles in early-stage gastric cancer (GC) lesions, focusing on the background gastric mucosa in patients who underwent endoscopic submucosal dissection. We aimed to reveal differences in gene expression profiles between patients with single and multiple GCs and to construct a scoring system for distinguishing between these two conditions. Using four biopsied specimens per patient, lesion-specific gene profiles were derived and analyzed using DNA microarrays. Overall, 21 genes exhibiting distinct expression profiles in relation to the background gastric mucosa were extracted. A scoring system was constructed by assigning weighted values to these 21 genes, with an optimal cutoff value of −2.574, yielding 85.7% sensitivity and specificity. The findings indicate that, compared to patients with a single GC, patients with multiple GCs have a more similar gene expression between the background gastric mucosa and the GC lesions.

## 1. Introduction

Gastric cancer (GC) is the fourth leading cause of cancer-related deaths worldwide, with approximately 769,000 fatalities annually [1]. The lifetime risk of GC-related mortality varies from region to region, ranging 0.32–0.63% in North America and 1.72–3.12% in East Asia [2]. In recent decades, the declining prevalence of *Helicobacter pylori* (*H. pylori*) infection has led to a significant decline in the incidence of GC. Endoscopic submucosal dissection (ESD) has become a widely adopted treatment modality for early-stage gastric cancer (EGC), as it preserves the stomach and thereby improves patients’ quality of life [3,4,5].

Synchronous or metachronous multiple GCs can be detected through follow-up esophagogastroduodenoscopy (EGD), typically performed every 6–12 months after ESD [6,7,8]. Submucosal invasive GCs may also be detected through follow-up EGDs after 1 year [8]. *H. pylori* infection-induced inflammation in the background gastric mucosa leads to DNA methylation in gastric epithelial cells, which is a key factor in GC development [9,10,11]. Intestinal metaplasia, characterized by increased levels of DNA methylation, is considered a precancerous lesion [12]. Therefore, increased DNA methylation levels in gastric epithelial cells are thought to correlate with a higher risk of metachronous GC [13].

Despite these insights, only a few marker genes have yet been identified to distinguish between single GC and multiple GCs that occur synchronously or metachronously in patients with EGC [14,15,16]. Our group has previously used a proprietary comprehensive gene expression analysis method to demonstrate the existence of disease-specific gene expression profiles [17]. This study aims to explore gene expression profiles that can distinguish between patients with a single GC and those with multiple GCs.

## 2. Materials and Methods

### 2.1. Patient Selection

Patients diagnosed with EGC and scheduled for ESD between June 2016 and March 2020 at Fukushima Medical University Hospital were included in the study. Written informed consent was obtained from each patient before participation, and the study was conducted in accordance with the Declaration of Helsinki and Japanese ethical guidelines. The Ethics Committee of Fukushima Medical University approved the study (approval no. 1953).

ESD was performed on lesions diagnosed as intramucosal GCs. Based on the number of eligible patients with EGC and the feasibility of recruitment, a maximum of 100 patients was set as the study limit over a 4-year period.

Patients were categorized into two groups: the “single GC group” and the “multiple GC group”. Multiple GCs were defined as the presence of additional lesions detected during ESD or a history of prior ESD for EGC. The single GC group included patients with a single lesion and no history of other GCs at the time of ESD. All patients were followed up until March 2023 (median follow-up period: 62 months, interquartile range [IQR]: 44–73 months).

### 2.2. Sample Collection

ESD for EGC was performed in accordance with the previously reported methods [18]. On the day of ESD, a single forceps biopsy specimen was obtained from the lesion before its removal. Simultaneously, biopsy specimens were collected from three nontumor mucosal sites: the greater curvature of the antrum, the lesser curvature of the gastric angle, and the greater curvature of the upper gastric body. These samples were immediately frozen with liquid nitrogen and preserved in tubes.

### 2.3. RNA Extraction and Gene Expression Profile Acquisition

Total RNA was extracted using ISOGEN (Nippon Gene Co., Ltd., Tokyo, Japan), following the manufacturer’s instructions. A custom array containing 14,400 synthetic polynucleotides (80 mers) (MicroDiagnostic, Tokyo, Japan), corresponding to human-derived transcripts, was used to print DNA microarrays for gene expression profiling on glass slides.

For patient-derived samples, SuperScript II (Invitrogen Life Technologies, Carlsbad, CA, USA) and cyclin 5-dUTP (Perkin-Elmer Inc., Boston, MA, USA) were used for synthesizing labeled cyclic DNA (cDNA) from 5 µg of total RNA, while SuperScript II and Cyanine 3-dUTP (Perkin-Elmer Inc., Kanagawa, Japan) were used for synthesizing the reference from 5 µg of Human Universal Reference RNA Type II (MicroDiagnostic, Tokyo, Japan). Hybridization was performed using the Labeling and Hybridization Kit (MicroDiagnostic, Tokyo, Japan).

Fluorescence intensity was measured using a GenePix 4000B Scanner (Axon Instruments, Inc., Union City, CA, USA), and the expression ratio (Cyanine-5/Cyanine-3 fluorescence intensity) was quantified. Further, the expression ratio was normalized by multiplying it by a factor derived using GenePix Pro 3.0 software (Axon Instruments, Inc., Tokyo, Japan). After log2-transformation of the ratios, they were processed using Excel (Microsoft, Bellevue, WA, USA) and the MDI gene expression analysis software package (MicroDiagnostic, Tokyo, Japan).

### 2.4. Comprehensive Gene Expression Analysis and Cluster Analysis

Genes with fluorescence intensity below the detection threshold in ≥7 of the 74 samples in the single GC group or in ≥3 of the 26 samples in the multiple GC group were excluded (Step 1-1). After calculating the mean of log2-transformed values for each group, genes with an absolute difference of 0.5 between the calculated mean and the log2-transformed values were selected (Step 1-2).

Genes with significant differences (*p* < 0.05) were extracted (Step 1-3) after a two-group comparison between the single and multiple GC groups. Expression View Pro (MicroDiagnostic, Tokyo, Japan) was used to perform the cluster analysis of the extracted genes (Step 1-4).

### 2.5. Risk Stratification, Comprehensive Gene Expression Analysis of Multiple Cancers, and Cluster Analysis

To ensure homogeneity between the single and multiple GC groups, patients who met any of the following conditions were excluded from the analysis: patients who had undergone gastrectomy after ESD, patients who had *H. pylori* eradication at an unknown age, patients in the single GC group diagnosed with metachronous GC, and patients in the multiple GC group who had *H. pylori* eradication before the age of 60. These excluded patients were considered to have a clinically low risk of GC, and their condition varied compared with other patients in the multiple GC group. The mean log2-transformed values (patient-specific log2-mean values) for each gene were calculated using the gene expression profiles obtained from one lesion and three nontumor mucosal samples. For the lesion samples, the value (patient mean relative value) was derived by subtracting the patient-specific log2-mean value from the log2-transformed value for each gene (Step 2-1). Genes with signals below the detection threshold in at least 3 samples of the 42 samples from the single GC group, or in at least 3 samples of the 13 samples from the multiple GC group, were excluded (Step 2-2). After calculating the means of the relative values for each group, genes with an absolute difference of ≥0.5 between the calculated mean values of the two groups were selected (Step 2-3). A two-group comparison was performed (*p* < 0.005), and Expression View Pro was used for cluster analysis (Step 2-4).

### 2.6. Construction of a Scoring System for Gene Expression Associated with Multiple Cancers and the Verification of Diagnostic Accuracy

The weighted mean relative values for the genes extracted in Step 2-3 were summed, and the “21-gene expression score” was calculated for each sample. For genes where “the mean value of the single GC group was found to be greater than or equal to the mean value of the multiple GC group”, the patient’s mean relative value was inverted by multiplying it by −1 (Step 2-5).

The genes were then sorted based on the 21-gene expression score to construct the scoring system (Step 2-6). Subsequently, the scatter plots, receiver operating characteristic (ROC) curves, and sensitivity/specificity curves for the single and multiple GC groups were generated (Step 2-7). The area under the curve (AUC) and optimal cutoff value were determined to maximize the sum of sensitivity and specificity (Step 2-8). The scoring system was validated using samples from patients excluded from the multiple GC group due to *H. pylori* eradication after the age of 60 and samples from patients with metachronous GC who did not undergo additional gastrectomy after ESD (Step 2-9).

### 2.7. Statistical Analysis

Cluster analysis was performed using the Euclidean distance and group mean method in Expression View Pro (MicroDiagnostic, Tokyo, Japan). SPSS Statistics 23 (SPSS, Inc., Chicago, IL, USA) was used to generate the ROC curve.

## 3. Results

### 3.1. Patient Characteristics

The clinical and pathological characteristics of the 100 patients with EGC included in this study are detailed in Table 1. Among these patients, 26 were classified into the multiple GC group, of which 14 had a history of GC, 10 had synchronous multiple GC lesions, and 2 had both synchronous and metachronous GCs lesions. The remaining 74 patients were categorized into the single GC group, of which 5 developed metachronous GC following ESD.

The median age of patients in the multiple GC group was significantly higher than that in the single GC group (81.5 vs. 70 years, *p* < 0.001), whereas the median tumor size in the multiple GC group was significantly smaller than in the single GC group (10 vs. 18.5 mm, *p* = 0.012). No significant differences were observed between the two groups regarding sex, histological GC type, tumor depth, lymphovascular invasion, *H. pylori* infection status, or endoscopic gastric atrophy.

### 3.2. Comprehensive Gene Expression Analysis and Clustering

**Figure 1:**
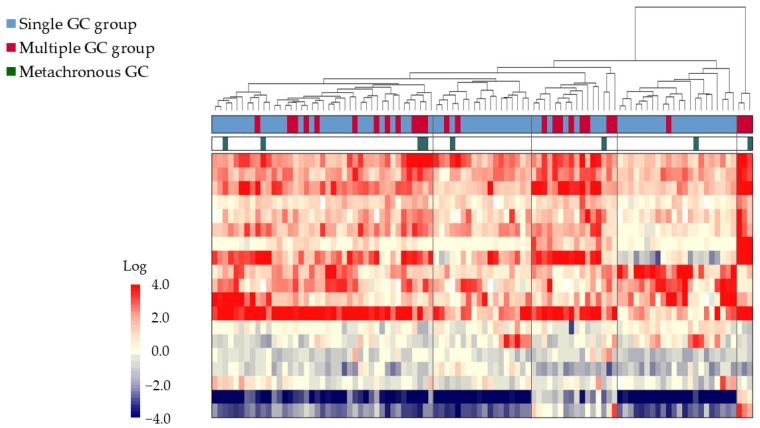
Comprehensive gene expression analysis of 100 GC biopsy samples obtained from patients undergoing ESD. A two-dimensional cluster analysis is represented as a heat map with a two-color gradient. The tree at the top of the heat map represents the relationships among the samples based on the dissimilarity coefficients. The single GC group is represented by the blue bars, while the multiple GC group is represented by the red bars, and metachronous cancers are represented by the green bars. The gene expression levels are represented by the red-to-blue gradient, with red denoting higher expression and blue denoting lower expression relative to the common reference. Samples were not distinctly classified into single or multiple GC groups. Abbreviations: ESD: endoscopic submucosal dissection; GC: gastric cancer; EGC: early-stage gastric cancer.

In the comprehensive gene expression analysis of all 100 patients, Step 1-1 narrowed the gene list to 10,741 genes. In Step 1-2, 28 genes were selected, and in Step 1-3, 19 genes were extracted. The results of the cluster analysis in Step 1-4 are shown in Figure 1. The 26 samples from the multiple GC group were scattered throughout the cluster, mixed with the single GC group samples, and did not form a distinct cluster.

### 3.3. Risk Stratification and Comprehensive Gene Expression Analysis

**Figure 2:**
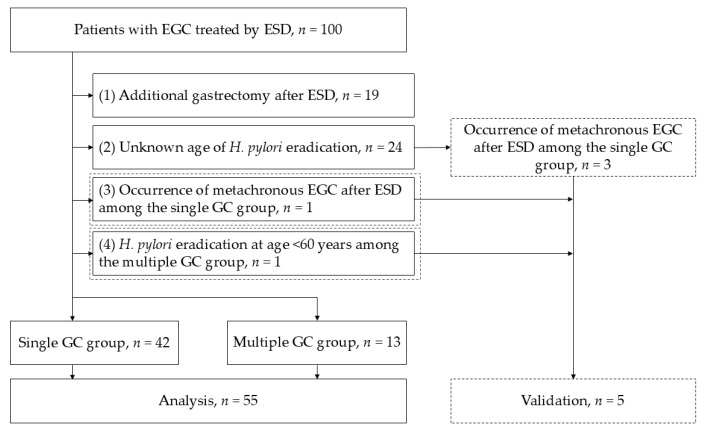
Flowchart illustrating the group homogeneity improvement process. Patients were stratified to ensure uniformity between the single and multiple GC groups. Of the 55 patients selected for further analysis, 42 patients were from the single GC group and 13 patients from the multiple GC group. Abbreviations: ESD: endoscopic submucosal dissection; GC: gastric cancer; EGC: early-stage gastric cancer; *H. pylori*: *Helicobacter pylori*.

To improve group homogeneity, 55 patients were selected for further analysis, of which 42 were from the single GC group and 13 from the multiple GC group (Figure 2). The median age of the multiple GC group was significantly higher (82.0 vs. 70.5 years, *p* = 0.016), but other clinical characteristics were comparable between the two groups (Table 2).

**Figure 3:**
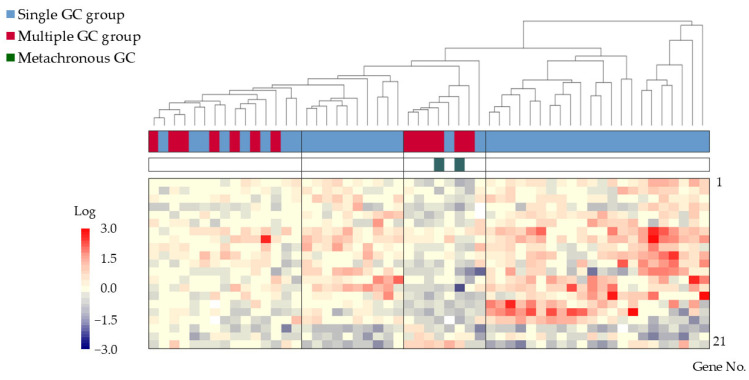
Cluster analysis of gene expression in 42 patients from the single GC group and 13 patients from the multiple GC group. Four clusters, with two clusters containing samples from both groups and two containing samples exclusively from the single GC group, were identified. Color bars and corresponding gene no. are listed from 1 to 21 in order from the top to the bottom. Abbreviation: GC: gastric cancer.

After narrowing the gene pool to 10,412 in Step 2-2, 142 genes with an absolute value difference ≥0.5 between the groups were extracted in Step 2-3. Statistically significant differences (*p* < 0.005, Step 2-4) were observed in 21 genes, which were then used for subsequent analyses (Table 3). Cluster analysis of these 21 genes revealed four distinct clusters, with two containing samples from both groups and two exclusively containing samples from the single GC group (Figure 3).

### 3.4. Gene Expression Scoring System

**Figure 4:**
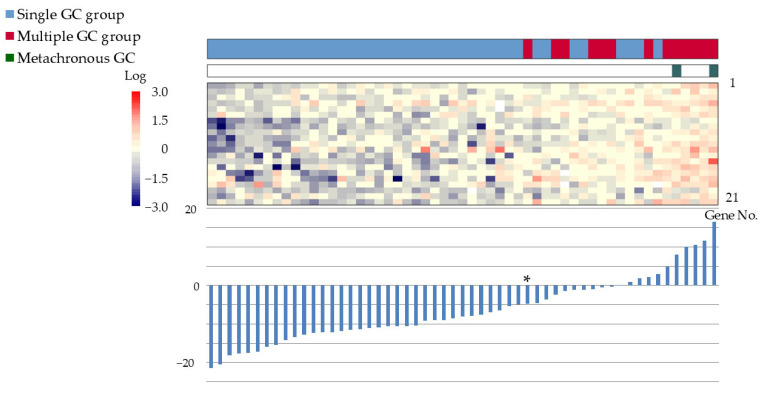
Gene expression scoring system for the multiple GC group. The expression scores of the 21 genes have been ranked in increasing order. Bar graphs display the scores, and heat maps depict the gene expression levels. The minimum score for the multiple GC group was −4.8613 (*). Color bars and corresponding gene no. are listed from 1 to 21 in order from the top to the bottom. Abbreviations: GC: gastric cancer; ESD: endoscopic submucosal dissection.

**Figure 5:**
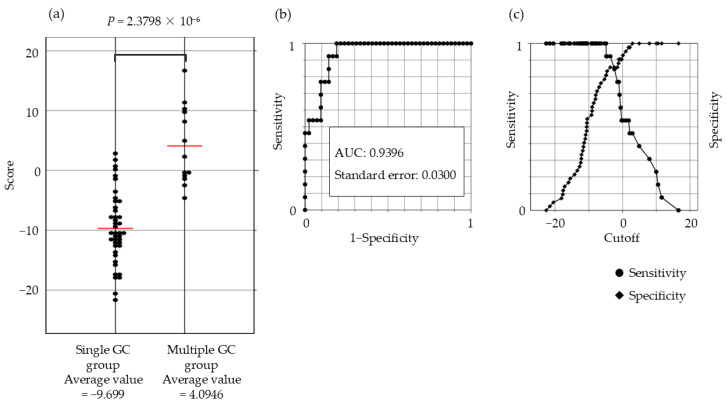
Scatter plot, ROC curve, and sensitivity–specificity curve for the 21-gene expression scoring system. (**a**) Significant differences in the scores between the single and multiple GC groups are evident from the scatter plot (*p* = 2.38 × 10^−6^) (**b**) Receiver operating characteristic curve (ROC) (**c**) The optimal cutoff value (−2.574) yielded a sensitivity and specificity of 85.7%. Abbreviations: GC: gastric cancer; ESD: endoscopic submucosal dissection; AUC: area under the curve.

**Figure 6:**
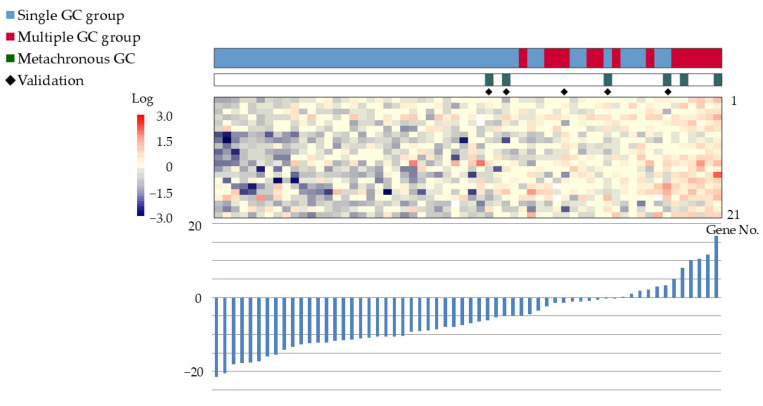
Validation of the gene expression scoring system using samples from five excluded patients. The validation samples scores exceeded −6.1203, confirming the system’s diagnostic accuracy. Color bars and corresponding gene no. are listed from 1 to 21 in order from the top to the bottom. Abbreviations: GC: gastric cancer; ESD: endoscopic submucosal dissection.

The gene expression scoring system is shown in Figure 4. The minimum score for the multiple GC group, applied to the 55 patients was −4.8613. The scatter plots, ROC curves, and sensitivity/specificity plots are depicted in Figure 5. The optimal cutoff value of −2.574 yielded a sensitivity and specificity of 85.7%, with an AUC of 0.94. Furthermore, validation using samples from five excluded patients yielded scores exceeding −6.1203, confirming the system’s diagnostic accuracy (Figure 6).

## 4. Discussion

This study presents a comprehensive analysis of gene expression profiles in Japanese patients with EGC lesions based on the background gastric mucosa, highlighting the differences between patients with single and multiple GCs. We also constructed a scoring system capable of distinguishing between these two groups. In this study, log-transformed values of mean gene expression levels in the GC lesion and background gastric mucosa were used to generate a relative profiling. Genes with expression levels differing between the cancerous area and the background gastric mucosa were extracted. A total of 21 genes with significantly different expression profiles between the single and multiple GC groups were extracted, and the resulting scoring system showed high sensitivity and specificity. The results indicate that patients with multiple GCs exhibit more similar gene expression levels between the GC lesion and background gastric mucosa compared with patients with a single GC. This insight is significant for understanding the biological differences between single GC and multiple GCs and may help improve post-ESD surveillance strategies.

In the present study, 21 differential genes, with distinct expression profiles stratified by background gastric mucosa, between the single and multiple groups were extracted. The function of each gene is detailed in the Supplementary Table S1. Several of the 21 extracted genes play critical roles in tumor biology. For example, WW domain-containing transcription regulator 1 (*WWTR1*) is a key regulator in the Hippo signaling pathway, which inhibits proliferation and promotes apoptosis to control organ size and tumor suppression [19,20]. However, overexpression of *WWTR1* has been associated with enhanced cancer growth and poor prognosis in GC [21,22]. Similarly, thrombospondin 1 (*THBS1*), a secreted protein, is responsible for inhibiting angiogenesis, regulating antitumor immunity, stimulating tumor cell migration, and modulating the activity of extracellular proteases and growth factors in the tumor microenvironment [23,24,25,26,27]. Other genes, such as the cell division cycle-associated protein (*CDCA*) 1-8 family, are thought to be involved in tumor progression, with high expression levels of *CDCA7* correlating with shorter disease-free survival in patients with GC [28]. Nuclear-Accumbens-Associated Kinase 2 (*NUAK2*) is involved in cell proliferation and survival, cell cycle regulation, and inhibition of apoptosis. Furthermore, it has been reported as a potential target for chemotherapy as an oncogene in several cancers [29,30,31,32].

Our scoring system showed lower expression levels for most of the 21 genes in the multiple GC group compared with those in the single GC group. This finding supports the hypothesis that gene expression in the background gastric mucosa and the cancerous lesion is more aligned in patients with multiple GCs. In our scoring system, the expression of 18 genes (gene No. 1 to 18 in Table 3) of the 21 genes, for which the mean value in the multiple GC group was lower than in the single GC group, was multiplied by −1. Higher scores in the system did not correspond to higher total expression levels of the 21 genes in the lesions of background gastric mucosa. Although these gene profiles are considered to be involved in the cascade of multiple synchronous or metachronous GC occurrences, this cross-sectional study could not reveal the detailed dynamics.

Despite the informative findings, this study has some limitations. First, the study was conducted on Japanese patients with existing *H. pylori* infection or a history of *H. pylori* infection. This narrowed the analysis to only 55 patients after improving group homogeneity. This small sample size could adversely affect the generalizability of the findings. Multicenter studies must be conducted to validate the results. Second, the relative complexity of the scoring system and stratification method may limit their clinical applicability. Several biomarkers have previously been reported for metachronous GC [14,15,16]. The scoring system in this study has the advantage of being able to distinguish not only metachronous GCs but also synchronous multiple GCs, but it is more complicated than existing biomarkers. Thus, it is necessary to further simplify the scoring system highlighted in this study for clinical usage. Third, possible factors affecting gene profiles, such as the histological subtype, age, and presence of *H. pylori* infection, were not examined as the sample size was too small. Fourth, the heterogeneous nature of GC lesions poses a challenge; differences in gene expression could arise from biopsy site variability. Finally, patient information in this study was obtained from electronic medical records in a retrospective manner, resulting in lack of other information such as alcohol and drinking history, diet type, and body mass index.

## 5. Conclusions

This study extracted genes associated with synchronous and metachronous multiple GCs and demonstrated the efficiency of a constructed scoring system to distinguish between multiple and single GC. These findings may provide valuable insights into the genetic basis of GC progression. However, multicenter studies are warranted to validate the results.

## Figures and Tables

**Table 1:** Clinicopathological characteristics of 100 patients with early-stage gastric cancer analyzed by comprehensive gene expression profiling.

	Single GC Group (n = 74)	Multiple GC Group (n = 26)	*p* Value
Age (years), median (P25−P75)	70.0 (65.0–80.0)	81.5 (72.5–85.0)	<0.001
Sex, *n* (%)			0.939
Male	49 (66.2)	17 (65.4)	
Female	25 (33.8)	9 (34.6)	
Follow-up duration (months), median (P25−P75)	62 (43.8–73.0)	60 (44.8–75.8)	0.838
History of GCs before enrollment, *n* (%)	N/A	16 (61.5)	N/A
Synchronous GCs during enrollment, *n* (%)	N/A	10 (38.5)	N/A
Metachronous GCs after protocol treatment, *n* (%)	5 (6.8)	3 (11.5)	0.425
*H. pylori* infection status, *n* (%) *			0.217
Negative	0	0	
Positive	31 (41.9)	7 (26.9)	
Eradicated	43 (58.1)	18 (69.2)	
Endoscopic gastric atrophy, *n* (%)			0.447
Closed type	9 (12.2)	1 (3.8)	
Open type	65 (87.8)	25 (96.2)	
Lesion			
Location, *n* (%)			0.342
Upper third	14 (18.9)	8 (30.8)	
Middle third	27 (36.5)	10 (38.5)	
Lower third	33 (44.6)	8 (30.8)	
Histological type, *n* (%) **			0.183
Pure differentiated	57 (77.0)	25 (96.2)	
Dominant differentiated	13 (17.6)	1 (3.8)	
Dominant undifferentiated	1 (1.4)	0	
Pure undifferentiated	3 (4.1)	0	
Tumor size (mm), median (P25−P75)	18.5 (12.0–28.0)	10.0 (7.5–23.5)	0.012
Tumor depth, *n* (%)			0.709
M	58 (78.4)	22 (84.6)	
SM1	15 (20.3)	4 (15.4)	
SM2	1 (1.4)	0	
Lymphovascular invasion, *n* (%)			
Ly	9 (12.2)	1 (3.8)	0.447
V	5 (6.8)	1 (3.8)	1.000

GC, gastric cancer; N/A, not applicable; *H. pylori: Helicobacter pylori*; M, intramucosal; SM1, minimal invasion of submucosa; SM2, ≥500 µm invasion of submucosa; Ly, lymphatic invasion; V, vascular invasion. * There was missing data (one case in *H. pylori* infection status). ** The differentiated type and the intestinal type have the same agreement, and the undifferentiated type and the diffuse type have the same agreement.

**Table 2:** Clinicopathological characteristics of 55 patients diagnosed with early-stage gastric cancer selected to ensure uniformity among all patients.

	Single GC Group (n = 42)	Multiple GC Group (n = 13)	*p* Value
Age (years), median (P25−P75)	70.5 (66.0–81.0)	82.0 (70.5–84.5)	0.016
Sex, n (%)			0.553
Male	27 (64.3)	8 (61.5)	
Female	15 (35.7)	9 (38.5)	
Follow-up duration (months), median (P25−P75)	63.5 (45.0–74.3)	59.0 (47.0–78.5)	0.905
History of GCs before enrollment, n (%)	N/A	7 (53.8)	N/A
Synchronous GCs during enrollment, n (%)	N/A	6 (46.2)	N/A
Metachronous GCs after protocol treatment, n (%)	1 (2.4)	2 (15.4)	0.136
*H. pylori* infection status, n (%)			0.622
Negative	0	0	
Positive	22 (52.4)	7 (53.8.9)	
Eradicated	20 (47.6)	6 (46.2)	
Endoscopic gastric atrophy, n (%)			0.672
Closed type	3 (7.1)	1 (7.7)	
Open type	39 (92.9)	12 (92.3)	
Lesion			
Location, n (%)			0.604
Upper third	11 (26.2)	4 (30.8)	
Middle third	16 (38.1)	3 (23.1)	
Lower third	15 (35.7)	6 (46.2)	
Histological type, n (%) *			0.427
Pure differentiated	37 (88.1)	13 (100)	
Dominant differentiated	4 (9.5)	0	
Dominant undifferentiated	1 (2.4)	0	
Pure undifferentiated	0	0	
Tumor size (mm), median (P25−P75)	16.5 (11.0–23.5)	15.5 (8.3–23.8)	0.647
Tumor depth, n (%)			0.562
M	40 (95.2)	12 (92.3)	
SM1	2 (4.8)	1 (7.7)	
SM2	0	0	
Lymphovascular invasion, n (%)			
Ly	2 (4.8)	0	0.580
V	0	0	N/A

GC, gastric cancer; *H. pylori: Helicobacter pylori*; M: intramucosal; SM1: minimal invasion of submucosa; SM2: ≥500 µm invasion of submucosa; Ly: lymphatic invasion; V: vascular invasion. * The differentiated type and the intestinal type have the same agreement, and the undifferentiated type and the diffuse type have the same agreement.

**Table 3:** Genes shown to distinguish single and multiple gastric cancer groups based on comprehensive gene expression analysis stratified by background gastric mucosa.

No	Symbol	Name	ID
1	*RBPMS*	mRNA for RBP-MS/type 3, complete cds.	D84109
2	*WWTR1*	WW domain-containing transcription regulator 1 (WWTR1), transcript variant 1, mRNA.	NM_015472
3	*CDCA7*	cDNA FLJ14722 fis, clone NT2RP3001621.	AK027628
4	*―*	cDNA FLJ36638 fis, clone TRACH2018950.	AK093957
5	*NUAK2*	NUAK family, SNF1-like kinase, 2 (NUAK2), mRNA.	NM_030952
6	*EDN1*	endothelin 1 (EDN1), transcript variant 1, mRNA.	NM_001955
7	*COL12A1*	collagen, type XII, alpha 1 (COL12A1), transcript variant long, mRNA.	NM_004370
8	*IGFBP7*	insulin-like growth factor binding protein 7 (IGFBP7), mRNA.	NM_001553
9	*DPYSL2*	dihydropyrimidinase-like 2 (DPYSL2), mRNA.	NM_001386
10	*THBS1*	thrombospondin 1 (THBS1), mRNA.	NM_003246
11	*FN1*	cellular fibronectin mRNA	M10905
12	*IGFBP5*	insulin-like growth factor binding protein 5 (IGFBP5), mRNA.	NM_000599
13	*SNHG18*	cDNA FLJ38512 fis, clone HCHON2000503.	AK095831
14	*CAMK2N1*	calcium/calmodulin-dependent protein kinase II, mRNA (cDNA clone MGC:22256 IMAGE:4703846), complete cds	BC020630
15	*MXRA5*	matrix-remodeling associated 5 (MXRA5), mRNA.	NM_015419
16	*TPH1*	tryptophan hydroxylase 1 (TPH1), mRNA.	NM_004179
17	*SCG2*	secretogranin II (SCG2), mRNA.	NM_003469
18	*―*	cDNA FLJ37762 fis, clone BRHIP2024347, weakly similar to GALECTIN-3.	AK095081
19	*ANO7*	cDNA FLJ32760 fis, clone TESTI2001812.	AK057322
20	*CA9*	carbonic anhydrase IX (CA9), mRNA.	NM_001216
21	*GPRC5B*	G protein-coupled receptor, family C, group 5, member B (GPRC5B), mRNA.	NM_016235

## Data Availability

Data is available upon request due to restrictions such as privacy or ethical considerations.

## Supplementary Materials

The following supporting information can be downloaded at: https://www.mdpi.com/article/10.3390/cancers17030464/s1, Supplementary Table S1: Function of Genes Extracted to Distinguish Single and Multiple Gastric Cancer Groups Based on Comprehensive Gene Expression Analysis Stratified by Background Gastric Mucosa.
