# Supplementary material for: A Single Center Study of Genes Involved in Synchronous and Metachronous Multiple Early-Stage Gastric Cancers in Japanese Patients with Current or Former Helicobacter pylori Infection
Source: Cancers (Basel). 2025 Jan 29;17(3):464. doi: 10.3390/cancers17030464 (PMC11815868; doi:10.3390/cancers17030464)
Supplement: Supplementary file 1 [file cancers-17-00464-s001.zip › cancers-3410806-supplementary.pdf]

Supplementary Table S1: Function of Genes Extracted to Distinguish Single and Multiple Gastric Cancer Groups Based on Comprehensive Gene Expression Analysis Stratified by Background Gastric Mucosa

No	Symbol	Name of the gene	ID	Functions
1	*RBPMS*	mRNA for RBP-MS/type 3, complete cds	D84109	RNA binding protein that mediates regulation of pre-mRNA alternative splicing (AS). Activates a splicing program associated with the differentiated contractile vascular smooth muscle cells by regulating the AS of numerous pre-mRNAs involved in actin cytoskeleton and focal adhesion machineries, thereby indicating a role in promoting a cell differentiated state.
2	*WWTR1*	WW domain containing transcription regulator 1 (WWTR1), transcript variant 1, mRNA	NM_015472	Transcriptional coactivator that functions as a downstream regulatory target in the Hippo signaling pathway, which plays a significant role in controlling organ size and tumor suppression by restricting proliferation and promoting apoptosis.
3	*CDCA7*	cDNA FLJ14722 fis, clone NT2RP3001621	AK027628	Participates in MYC-mediated cell transformation and apoptosis. Induces anchorage-independent growth and clonogenicity in lymphoblastoid cells. Insufficient to induce tumorigenicity when overexpressed; however, contributes to MYC-mediated tumorigenesis. Might play a role as a transcriptional regulator.
4	—	cDNA FLJ36638 fis, clone TRACH2018950	AK093957	Unknown
5	*NUAK2*	NUAK family, SNF1-like kinase, 2 (NUAK2), mRNA	NM_030952	Protects cells from CD95-mediated apoptosis and plays a role in the increased motility and invasiveness of the CD95-activated tumor cells.
6	*EDN1*	Endothelin 1 (EDN1), transcript variant 1, mRNA	NM_001955	Endothelium-derived vasoconstrictor peptide. Promotes mesenteric arterial wall remodeling through the activation of ROCK signaling and subsequent colocalization of NFATC3 with F-actin filaments.
7	*COL12A1*	Collagen, type XII, alpha 1 (COL12A1), transcript variant long, mRNA	NM_004370	Type XII collagen that interacts with the type I collagen-containing fibrils. The COL1 domain could be associated with the surface of the fibrils, and the COL2 and NC3 domains might be localized in the perifibrillar matrix.

8	*IGFBP7*	Insulin-like growth factor binding protein 7 (IGFBP7), mRNA	NM_001553	Stimulates prostacyclin (PGI2) production and cell adhesion. Functions as a ligand for CD93 to play a role in angiogenesis.
9	*DPYSL2*	Dihydropyrimidinase-like 2 (DPYSL2), mRNA	NM_001386	Involved in neuronal development and polarity as well as in axon growth and guidance, neuronal growth cone collapse, and cell migration. Required for signaling by class 3 semaphorins and subsequent remodeling of the cytoskeleton. May play a role in endocytosis.
10	*THBS1*	Thrombospondin 1 (THBS1), mRNA	NM_003246	Multifunctional. Plays a role in inflammation, angiogenesis, wound healing, reactive oxygen species signaling, nitrous oxide signaling, apoptosis, senescence, aging, cellular self-renewal, stemness, and cardiovascular and metabolic homeostasis. Also involved in inducing apoptosis of podocytes in response to elevated free fatty acids, acting via CD36.
11	*FN1*	Cellular fibronectin mRNA	M10905	Fibronectins are involved in cell adhesion, cell motility, opsonization, wound healing, and maintenance of cell shape.
12	*IGFBP5*	Insulin-like growth factor binding protein 5 (IGFBP5), mRNA	NM_000599	Multifunctional protein that plays a vital role in regulating the availability of IGFs to their receptors and thus regulates IGF-mediated cellular processes including proliferation, differentiation, and apoptosis in a cell-type specific manner.
13	*SNHG18*	cDNA FLJ38512 fis, clone HCHON2000503	AK095831	Unknown
14	*CAMK2N1*	Calcium/calmodulin-dependent protein kinase II, mRNA (cDNA clone MGC:22256 IMAGE:4703846), complete cds	BC020630	Modulates blood pressure and vascular reactivity by regulating the CAMK2 activity in addition to the regulation of left ventricular mass. Mediates the NLRP3 inflammasome in cardiomyocytes by acting as an inhibitor of the MAPK14/p38 and MAPK8/JNK pathways, thereby regulating ventricular remodeling and cardiac rhythm post-myocardial infarction.
15	*MXRA5*	Matrix-remodeling associated 5 (MXRA5), mRNA	NM_015419	In the kidney, carries out anti-inflammatory and antifibrotic functions by limiting the induction of chemokines, fibronectin, and

				collagen expression in response to TGB1 and pro-inflammatory stimuli.
16	*TPH1*	Tryptophan hydroxylase 1 (TPH1), mRNA	NM_004179	Oxidizes L-tryptophan to 5-hydroxy-l-tryptophan in the rate-determining step of serotonin biosynthesis.
17	*SCG2*	Secretogranin II (SCG2), mRNA	NM_003469	Neuroendocrine protein belonging to the granin family that regulates the biogenesis of secretory granules.
18	—	cDNA FLJ37762 fis, clone BRHIP2024347, weakly similar to GALECTIN-3	AK095081	Unknown
19	*ANO7*	cDNA FLJ32760 fis, clone TESTI2001812	AK057322	Has calcium-dependent phospholipid scramblase activity; scrambles phosphatidylserine, phosphatidylcholine, and galactosylceramide.
20	*CA9*	Carbonic anhydrase IX (CA9), mRNA	NM_001216	Acts as a catalyst in the interconversion between carbon dioxide and water and the dissociated ions of carbonic acid.
21	*GPRC5B*	G-protein-coupled receptor, family C, group 5, member B (GPRC5B), mRNA	NM_016235	G-protein coupled receptor involved in the regulation of cell volume.
